# Supplementary figures and images for: Properties of a cryptic lysyl oxidase from haloarchaeon Haloterrigena turkmenica
Source: PeerJ. 2019 Apr 5;7:e6691. doi: 10.7717/peerj.6691 (PMC6452851; doi:10.7717/peerj.6691)

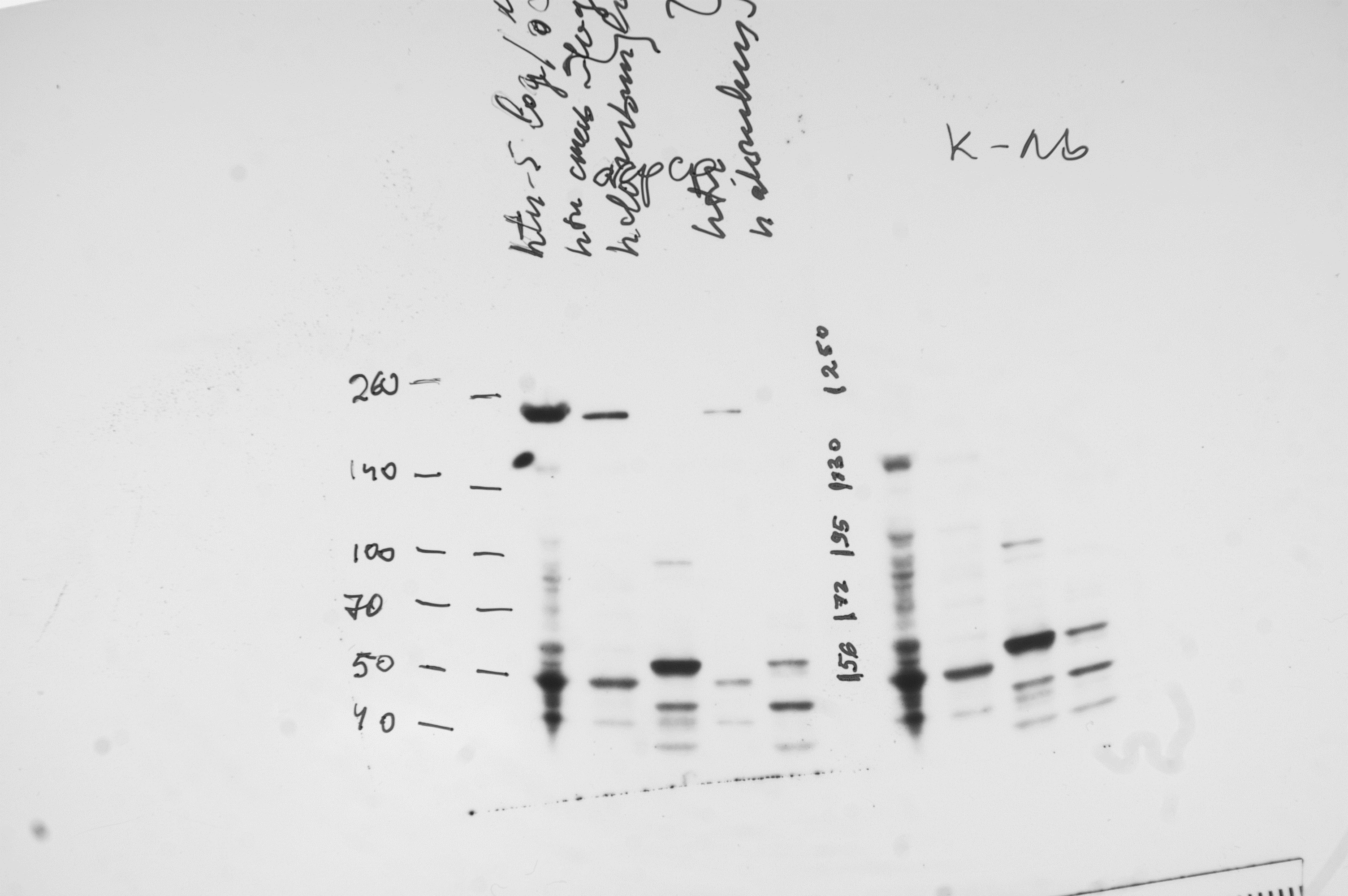

Supplement: Supplemental Information 1 — Uncropped and unedited film of western blotting used for Fig. 4. [file peerj-07-6691-s001.png]
