# Supplementary material for: Properties of a cryptic lysyl oxidase from haloarchaeon Haloterrigena turkmenica
Source: PeerJ. 2019 Apr 5;7:e6691. doi: 10.7717/peerj.6691 (PMC6452851; doi:10.7717/peerj.6691)
Supplement: Supplemental Information 2 [file peerj-07-6691-s002.docx]

| **pH** | 5,8 | 6,0 | 6,1 | 6,2 | 6,3 | 6,4 | 6,5 |
| --- | --- | --- | --- | --- | --- | --- | --- |
| **-BAPN** | 10569 | 11068 | 12037 | 16301 | 11455 | 9024 | 8394 |
| **+BAPN** | 7325 | 6920 | 6770 | 7010 | 5479 | 4528 | 4521 |
| ∆ | 3244 | 4148 | 5267 | 9291 | 5976 | 4496 | 3873 |

**Effect of different pH values on refolding of HTU-LOX.** Arbitrary units of HTU-LOX activity, measured after dialysis at different pH. BAPN – 3-aminopropionitrile.

| **Cu^2+^ concentration, mcM** | --- | 0.1 | 1 | 10 |
| --- | --- | --- | --- | --- |
| **- BAPN** | 18288 | 28023 | 35576 | 30847 |
| **+ BAPN** | 3144 | 3621 | 4041 | 2345 |
| **∆** | 15144 | 24402 | 31535 | 28502 |

**Effect of different Cu^2+^ concentration during dialysis on protein activity.** Arbitrary units of resorufin fluorescence corresponding to peroxide production by HTU-LOX amine oxidase activity, measured after dialysis in buffer with different Cu^2+^ concentrations.

| Amine Substrates | K_m_, mM | V_max_*10^-3^ (nmole*s^-^1*mg^-1^) |
| --- | --- | --- |
| Histamine | 0.0240±0,0034 | 129.631±25,308 |
| Polymyxin | 0.041±0,006 | 59.406±22,938 |
| Amikacin | 0.136±0,089 | 128.409±10,404 |
| Capreomycin | 1.018±0,085 | 369.418±5,313 |

**Explanation of primer sequences used in this study:**

HTU-AADN GGACGGATCCGCGGCTGACAACCCGTTCACA

HTU-QVED CGCAGGATCCCAAGTTGAGGACCAGCCGGAGGTCAAC

HTU-C TAGGAAGCTTAGCAGGAAGGGGGTTTCACAT

ARCH16S-F2 TCCGGTTGATCCYGCCGGA

ARCH16S-R934 GTGCTCCCCCGCCAATTC

Primers HTU-AADN and HTU-QVED contain *Bam*H I sites and were used as direct primers from cloning HTU-AA and HTU-QV fragments, respectively. Reverse primer HTU-C containing a *Hin*d III site was the same in both PCRs. Primers ARCH16S-F2 and ARCH16S-R934 were used for PCR and sequencing of 16S RNA genes.


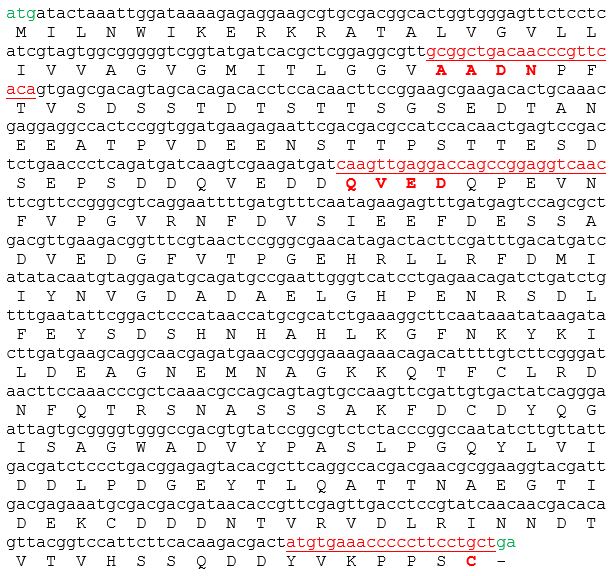


***Nucleotide and amino acid sequences of H. turkmenica lysyl oxidase ORF.*** *Red – position of primer sequences used in this study.*


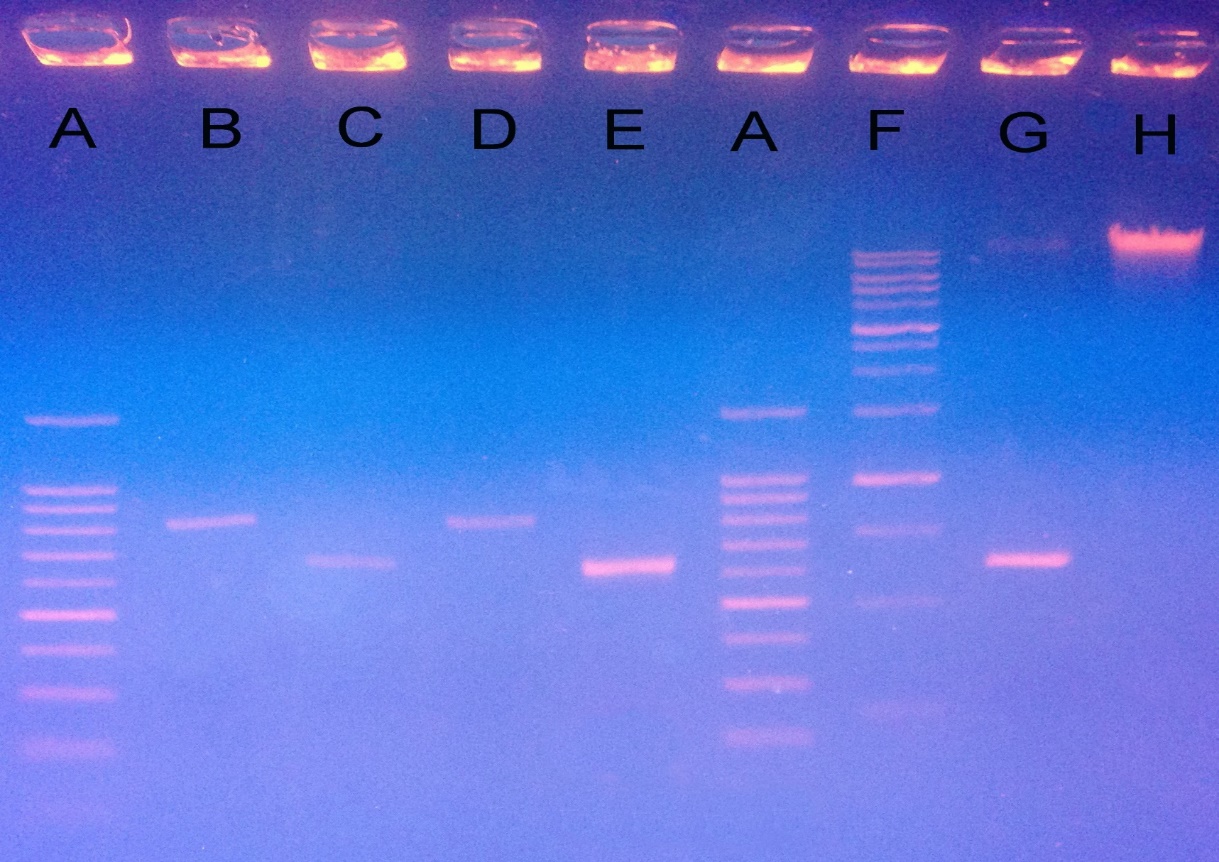


**Agarose gel electrophoresis of PCR products.** A, 100 bp DNA marker ladder; B, *Taq* polymerase, HTU-AADN; C, *Taq* polymerase, HTU-QVED; D, Phusion polymerase, HTU-AADN; E, Phusion polymerase, HTU-QVED; F, 1 kb DNA ladder; G, Phusion polymerase, HTU-QVED; H, *H. turkmenica* VKM B-1732 genomic DNA.

**Expression of both HTU-AA and HTU-QV fragments simultaneously**

**
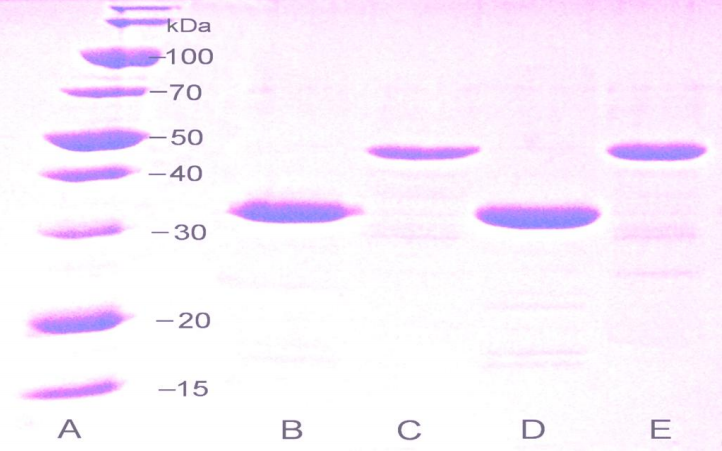
**

**Electrophoretic analysis of purified recombinant proteins HTU-AA and HTU-QV, fragments of *H. turkmenica* LOX.** A , molecular weight marker proteins; B, D, protein HTU-QV; C, E, protein HTU-AA.





**Immunoblotting detection of full-length HTU-LOX in *E. coli* with anti-HTU-LOX antibodies.** SDS-PAGE in Any kD gradient gel (Bio-Rad, USA). 1, 2 – chemiluminescence of bound HRP-labeled antibodies (affinity purified); molecular weight markers to the right. 1 – proteins from *E. coli* producing an unrelated recombinant protein (human paraoxonase 1212) as a negative control, 2 – *E. coli* with expression of a full length HTU-LOX. Proteins were


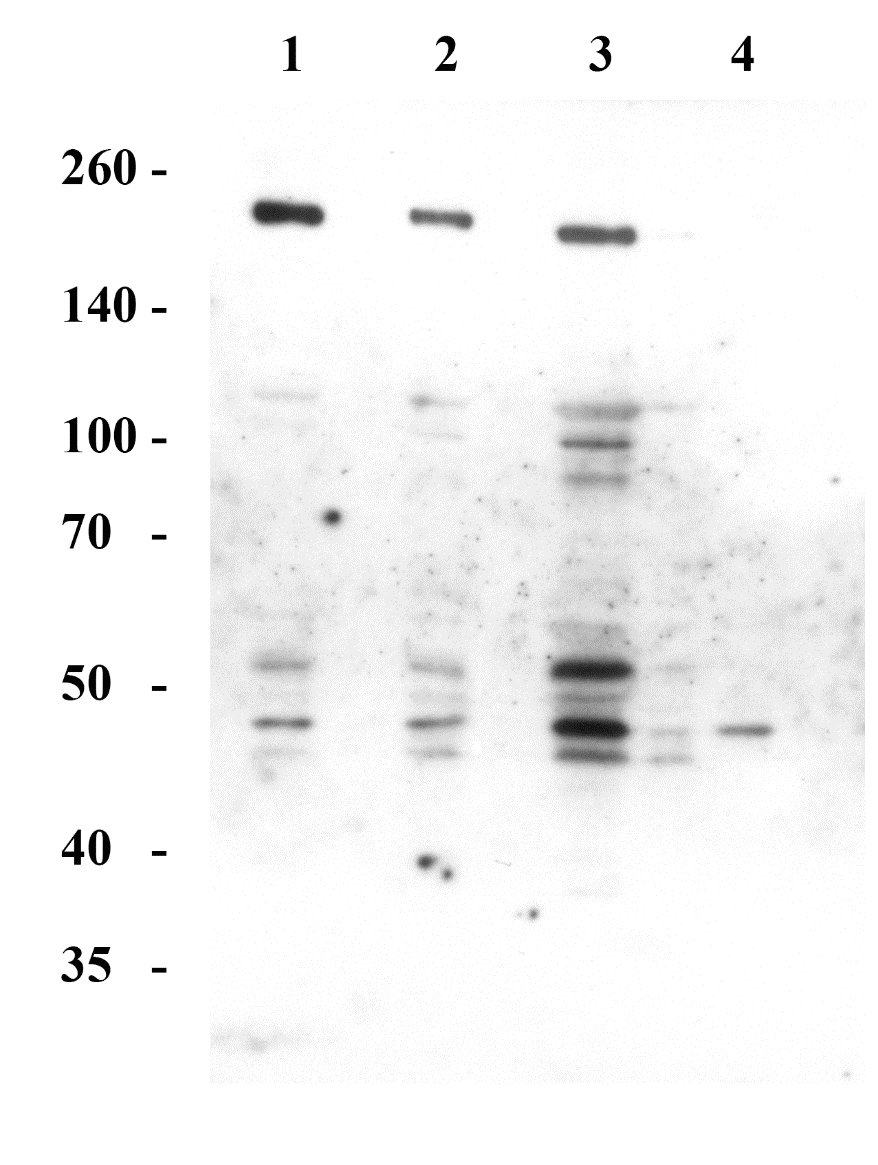


**Immunoblotting detection of lysyl oxidase in *H. turkmenica* cells with anti-HTU-LOX antibodies.** SDS-PAGE in 8% gel. 1, 2, 3, 4 – chemiluminescence of bound HRP-labeled antibodies (crude serum, not affinity purified); molecular weight markers on the left. 1 – *H. turkmenica* cells grown in IAO medium to log-phase (OD_600_ 0.3); 2 – cells in stationary phase (OD_600_ 1.2); 3 – cells grown to stationary phase were incubated at room temperature (around 25°C) for 7 days; 4 - 3 – cells were incubated at room temperature (around 25°C) for one month in saturated medium with precipitating salt.

**SUPPLEMENTARY TABLE**

**Lack of detectable amine oxidase activity of full-length HTU-LOX protein expressed in *E. coli* and in *Haloterrigena* proteins**

| Sample | Activity,  nmole*mg^-1^*sec^-1^ |
| --- | --- |
| *E. coli* crude cell lysate, full-length HTU-LOX | <0.0001 |
| *E. coli* crude cell lysate, HTU-AA | 0.0053 |
| Purified and refolded HTU-QV | 0.079 |
| *Haloterrigena turkmenica*, secreted proteins | <0.0001 |
| *Haloterrigena turkmenica,* crude cell lysate | <0.0001 |

XL-1 *E. coli* were induced with IPTG as described in Results, cells pelleted, lysed using freeze-thawing followed by homogenization by bead-beating (water, 0.1 mm glass beads) and extensively dialyzed against the acetate folding buffer. Fresh *Haloterrigena* cells were centrifuged and lysed in water similarly to the procedure described in the immunoblotting section (before the addition of TCEP and other components) and extensively dialyzed against the acetate folding buffer. Secreted proteins fraction has been obtained by ultrafiltration and washing of the cleared *Haloterrigena* culture supernatant on a 10000 MWL filter (Amicon, USA). For activity measurement, 2 mM histamine has been chosen here as amine substrate. <0.0001 indicates values below detection limit with this assay.
